# Antibacterial Activity and Molecular Docking Studies of a Selected Series of Hydroxy-3-arylcoumarins

**DOI:** 10.3390/molecules24152815

**Published:** 2019-08-01

**Authors:** Maria Barbara Pisano, Amit Kumar, Rosaria Medda, Gianluca Gatto, Rajesh Pal, Antonella Fais, Benedetta Era, Sofia Cosentino, Eugenio Uriarte, Lourdes Santana, Francesca Pintus, Maria João Matos

**Affiliations:** 1 Department of Medical Sciences and Public Health, University of Cagliari, Cittadella Universitaria, 09042 Monserrato, Italy; 2 Department of Electrical and Electronic Engineering, University of Cagliari, Via Marengo 2, 09123 Cagliari, Italy; 3 Department of Sciences of Life and Environment, University of Cagliari, Cittadella Universitaria, 09042 Monserrato, Italy; 4 Department of Biomedical Sciences, University of Cagliari, Cittadella Universitaria, 09042 Monserrato, Italy; 5 Department of Organic Chemistry, Faculty of Pharmacy, University of Santiago de Compostela, 15782 Santiago de Compostela, Spain; 6 Instituto de Ciencias Químicas Aplicadas, Universidad Autónoma de Chile, Santiago 7500912, Chile

**Keywords:** hydroxy-3-arylcoumarins, Perkin–Oglialoro reaction, antibacterial activity, molecular docking

## Abstract

Antibiotic resistance is one of the main public health concerns of this century. This resistance is also associated with oxidative stress, which could contribute to the selection of resistant bacterial strains. Bearing this in mind, and considering that flavonoid compounds are well known for displaying both activities, we investigated a series of hydroxy-3-arylcoumarins with structural features of flavonoids for their antibacterial activity against different bacterial strains. Active compounds showed selectivity against the studied Gram-positive bacteria compared to Gram-negative bacteria. 5,7-Dihydroxy-3-phenylcoumarin (compound **8**) displayed the best antibacterial activity against *Staphylococcus aureus* and *Bacillus cereus* with minimum inhibitory concentrations (MICs) of 11 μg/mL, followed by *Staphylococcus aureus* (MRSA strain) and *Listeria monocytogenes* with MICs of 22 and 44 μg/mL, respectively. Moreover, molecular docking studies performed on the most active compounds against *Staphylococcus aureus* tyrosyl-tRNA synthetase and topoisomerase II DNA gyrase revealed the potential binding mode of the ligands to the site of the appropriate targets. Preliminary structure–activity relationship studies showed that the antibacterial activity can be modulated by the presence of the 3-phenyl ring and by the position of the hydroxyl groups at the coumarin scaffold.

## 1. Introduction

Antibiotic resistance is one of the leading public health concerns of this century [1,2], mainly due to the emergence, spread, and persistence of multidrug-resistant (MDR) bacteria, informally named as “superbugs”, which cause infections that fail to respond to conventional treatments. The increasingly widespread use and misuse of antibiotics in animals and humans, as well as the lack of innovation in antibiotic research (decline in the number of new antibiotic classes), are among the leading causes of the development and spread of antimicrobial resistance [3]. Policies to control the inappropriate and irrational use of antibiotics are urgently needed, as is the development of new chemical entities as antibacterial agents [4]. 

A group of MDR bacteria collectively known as “ESKAPE”, which includes Gram-positive and Gram-negative species (*Enterococcus faecium*, *Staphylococcus aureus*, *Klebsiella pneumoniae*, *Acinetobacter baumannii*, *Pseudomonas aeruginosa*, and *Enterobacter* spp.), are frequently isolated in hospital environments, where they are responsible for the majority of nosocomial infections [5]. In particular, Gram-positive bacteria have predominantly developed resistance to all the available antibiotics and pose a serious problem not only in hospitals but also for the general population [6,7]. Infections of methicillin-resistant *Staphylococcus aureus* (MRSA) are of particular concern [8].

Several recent reports associate antibiotic resistance with oxidative stress [9,10]. Therefore, molecules presenting dual activity as antibacterial and antioxidant can be attractive candidates [9].

Tyrosyl-tRNA synthetase represents an attractive target enzyme for finding new antibacterial agents [11]. It belongs to the aminoacyl-tRNA synthetases (aaRSs) and is responsible for catalyzing the covalent binding of amino acids to their respective tRNA to form charged tRNA. Thus, inhibition of aaRSs affects cell growth due to their key role in the protein biosynthesis process. Topoisomerase II DNA gyrase is another target enzyme, and coumarins have proven to be one of the most studied families of inhibitors of this enzyme [12]. DNA topoisomerases catalyze changes in the topology of DNA and are essential for cell survival. DNA gyrase is a type II topoisomerase that can introduce negative supercoils into DNA by ATP consumption. It is essential in all bacteria but is absent from higher eukaryotes, making it an attractive target for antibacterial agents [13].

**Figure 1:**
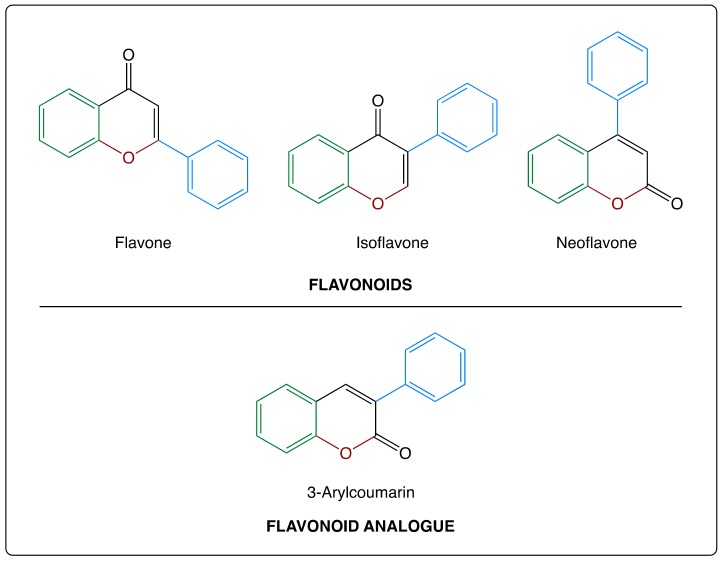
Chemical structure of some flavonoids and a flavonoid analogue (3-arylcoumarin).

Coumarins represent an important family of naturally occurring and/or synthetic compounds that are well known for their pharmacological activities [14,15]. 4-Arylcoumarins or neoflavones, shown in Figure 1, are examples of naturally occurring flavonoids [16] with antioxidant and antibacterial activities [17,18].

Matos et al. have already reported some simple coumarins and 3-phenylcoumarins as interesting antibacterial agents for human use, in particular against clinical isolates of *S. aureus* [19,20]. The 3-(3′-methylphenyl)-6-nitrocoumarin proved to be the best compound of that series, presenting a minimum inhibitory concentration (MIC) for *S. aureus* of 8 μg/mL. 

Taking this into account, we investigated a series of 3-arylcoumarins with structural features of flavonoids and evaluated their antibacterial and antioxidant activity. The aim of our work was to understand how the structural modification of these compounds could affect the biological activities. 

For the current study, different chemical features were explored in order to increase the chemical space and the potential interaction profile with the targets. 

Herein, a series of 3-arylcoumarins were synthesized and evaluated against four Gram-positive and three Gram-negative strains. Moreover, their antioxidant properties were considered. Molecular docking studies using tyrosyl-tRNA synthetase and topoisomerase II DNA gyrase from *S. aureus* were also performed to better understand the mechanism of action of these molecules.

## 2. Results and Discussion 

**Scheme 1:**
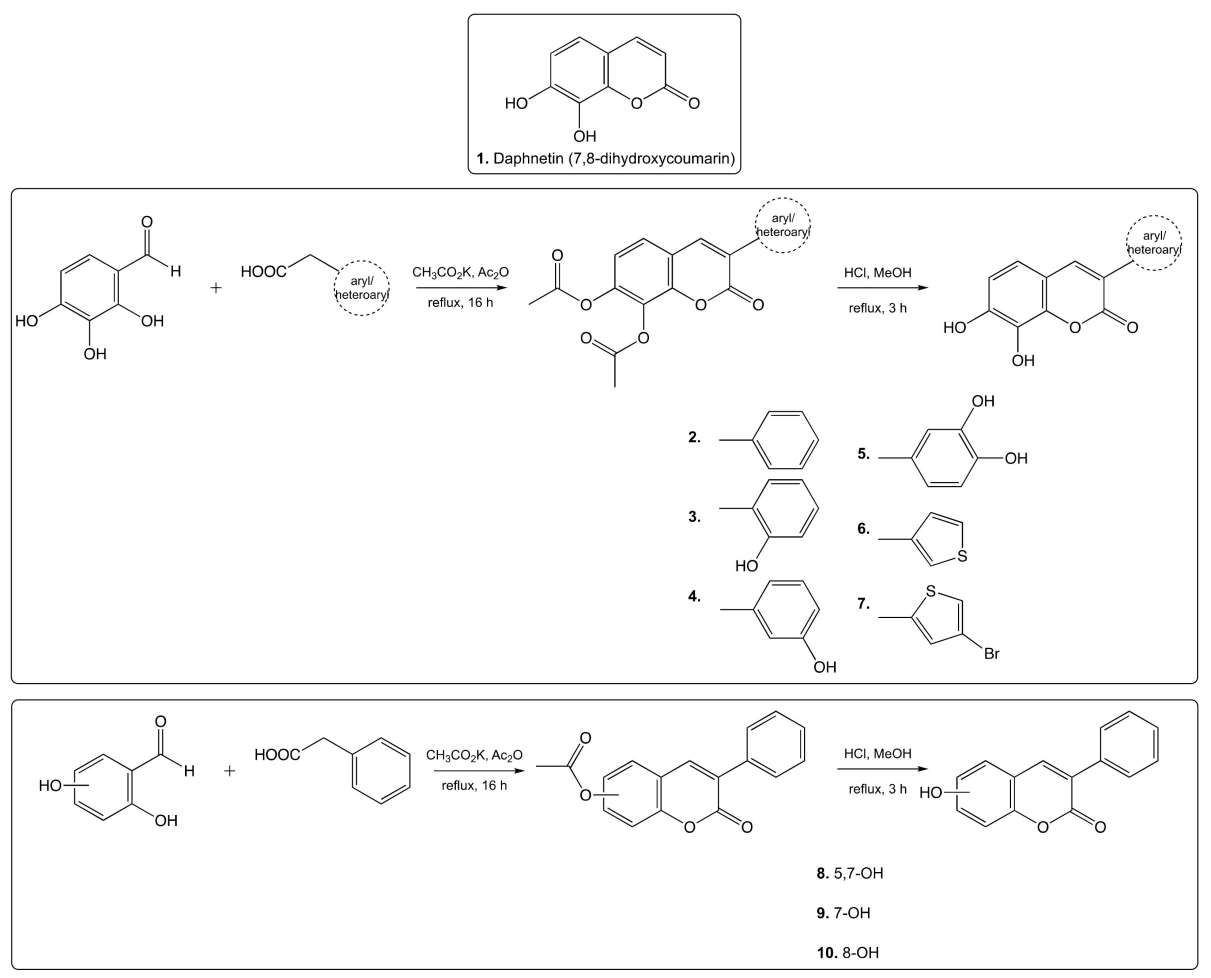
Synthetic routes and chemical structures of compounds **1**–**10**.

Compounds **2**–**10** are known and were synthesized via a two-step Perkin–Oglialoro reaction (Scheme 1). Different commercially available *ortho*-hydroxybenzaldehydes and aryl/heteroarylacetic acids were condensed in the presence of potassium acetate (CH_3_CO_2_K) in acetic anhydride (Ac_2_O) under reflux for 16 h to afford the acetoxy-3-aryl/heteroarylcoumarins. This step involves sequential acetylation of the hydroxyl groups and pyrone ring closure in a single-pot operation. Further on, the hydrolysis of the obtained acetoxy derivatives, in the presence of aqueous hydrochloric acid (HCl) and methanol (MeOH) under reflux for 3 h, afforded the hydroxyl substituted 3-aryl/heteroarylcoumarins **2**–**10**. Compound **1** (daphnetin) is commercially available from Sigma-Aldrich.

The synthesized compounds were screened for antibacterial activity against different bacterial strains, including both Gram-positive and Gram-negative bacteria, and the results are reported in Table 1. 

Gram-negative bacteria proved to be the less sensitive strains, being resistant to all the compounds at the maximum tested concentrations (500/350 µg/mL). 

The lack of activity or the lower susceptibility of the Gram-negative versus Gram-positive bacteria may be due to the variation in their cell wall structure. In fact, Gram-negative bacteria possess an outer lipidic membrane that restricts the access to the periplasm by acting as efficient selective permeation barrier [21]. 

Compounds **2**–**5** and **8** were found to exhibit antibacterial activity against Gram-positive bacteria but to different extents. Compound **8** was the most active, followed by compound **2**, which showed an interesting effect against *L. monocytogenes* (MIC = 125 µg/mL) and presented a slightly better activity against the two *S. aureus* strains tested (both MICs = 62.5 µg/mL). The highest activity of compound **2** was detected against *B. cereus*, with a remarkable MIC of 15.6 µg/mL. A similar activity was obtained for compound **8** against *B. cereus*, with a MIC of 11 µg/mL. This compound also showed the highest antibacterial potential against *L. monocytogenes* (MIC = 44 µg/mL), *S. aureus* ATCC 25923 (MIC = 11 µg/mL), and MRSA TN2A strain (MIC = 22 µg/mL). These MIC values are comparable with those observed in previous studies describing the antibacterial activity of coumarin derivatives against Gram-positive bacteria, including *S.* aureus [22,23,24]. Members of this species are considered opportunistic pathogens responsible for a broad spectrum of diseases ranging from superficial skin infections to systemic infections, with the ability to acquire resistance to any antibiotic [25].

An in-depth analysis of structure–activity relationship was performed and indicated that the simultaneous presence of a phenyl ring at position 3 and two hydroxy groups at positions 5 and 7 of the coumarin scaffold (compound **8**) improved the antibacterial activity of the coumarin derivatives. In fact, compound **1** (7,8-dihydroxycoumarin), which does not have any aromatic substituent at position 3 of the coumarin ring and is the simplest compound of the series, did not exert any antibacterial activity at the highest tested concentration (500 µg/mL). The same results were obtained for compounds **6** and **7**, which differed from compound **1** on the presence of a thiophenyl ring at position 3 of the coumarin structure (Scheme 1). Also, all the studied 3-phenyl derivatives (compounds **2**–**5**) were found to be active, with compound **2** the most active among them, followed by **4** and **3**, while compound **5** was the least active from this group. Considering the structure of the compounds within this series, it seems that the presence of hydroxyl groups on the 3-phenyl ring decreases the antibacterial activities. The presence of two hydroxyl groups on the fused benzo ring of the coumarin scaffold and the contemporary presence of a phenyl ring at position 3 (compounds **2**–**5** and **8)** seems to be essential for antibacterial activity. Compounds **9** and **10** with a single hydroxyl group exhibited no activity when compared to the dihydroxycoumarin derivative **8**. To further elucidate the importance of the hydroxyl group substitutions in the phenyl ring, compound **8** was tested, and it proved to be the best compound of the studied series. Thus, the two hydroxyl groups at positions 5 and 7 of the coumarin ring increased the antibacterial activities. These positions can be further studied in order to optimize the profile of this compound as antibacterial.

Some examples have been reported of compounds combining in one single structure with both antioxidant and antibacterial activities as two synergistic properties [9]. Taking this into account, the most active compounds were evaluated for their antioxidant properties. The results are shown in Table 2. 

All the studied compounds had redox properties, which would allow them to act as antioxidants. Among them, compound **2** showed antioxidant activity comparable to Trolox, and compounds **5** and **8** had EC_50_ values significantly better than that of the positive control.

These results led us to consider compound **8** as a promising compound with dual antibacterial and antioxidant activity and therefore as a potential candidate to serve as an antibiotic.

The molecular properties of the most active compounds, namely, the dihydroxy-substituted derivatives **2** and **8** as well as **9**, which contains only one hydroxyl group, were predicted using Swiss-ADME web server [27] against the known antibiotic, ampicillin (Table 3). 

Compounds **2** and **8** shared similar physicochemical and pharmacokinetic properties as they had an identical number of atoms and differed only in the hydroxyl group position within the coumarin scaffold. On the other hand, compound **9** lacked one hydroxyl group compared to compounds **2** and **8**. This difference was reflected in the lower value of polar surface area and in the number of hydrogen bond acceptor and donor atoms. The coumarin-derived compounds were less flexible with respect to ampicillin, evident from a lower number of rotatable bonds. Furthermore, compounds **2**, **8**, and **9** displayed high gastrointestinal absorption (GA) and blood–brain barrier (BBA) permeation properties, which are absent in ampicillin. All the molecules exhibited druglikeness characteristics according to Lipinski rules. 

**Figure 2:**
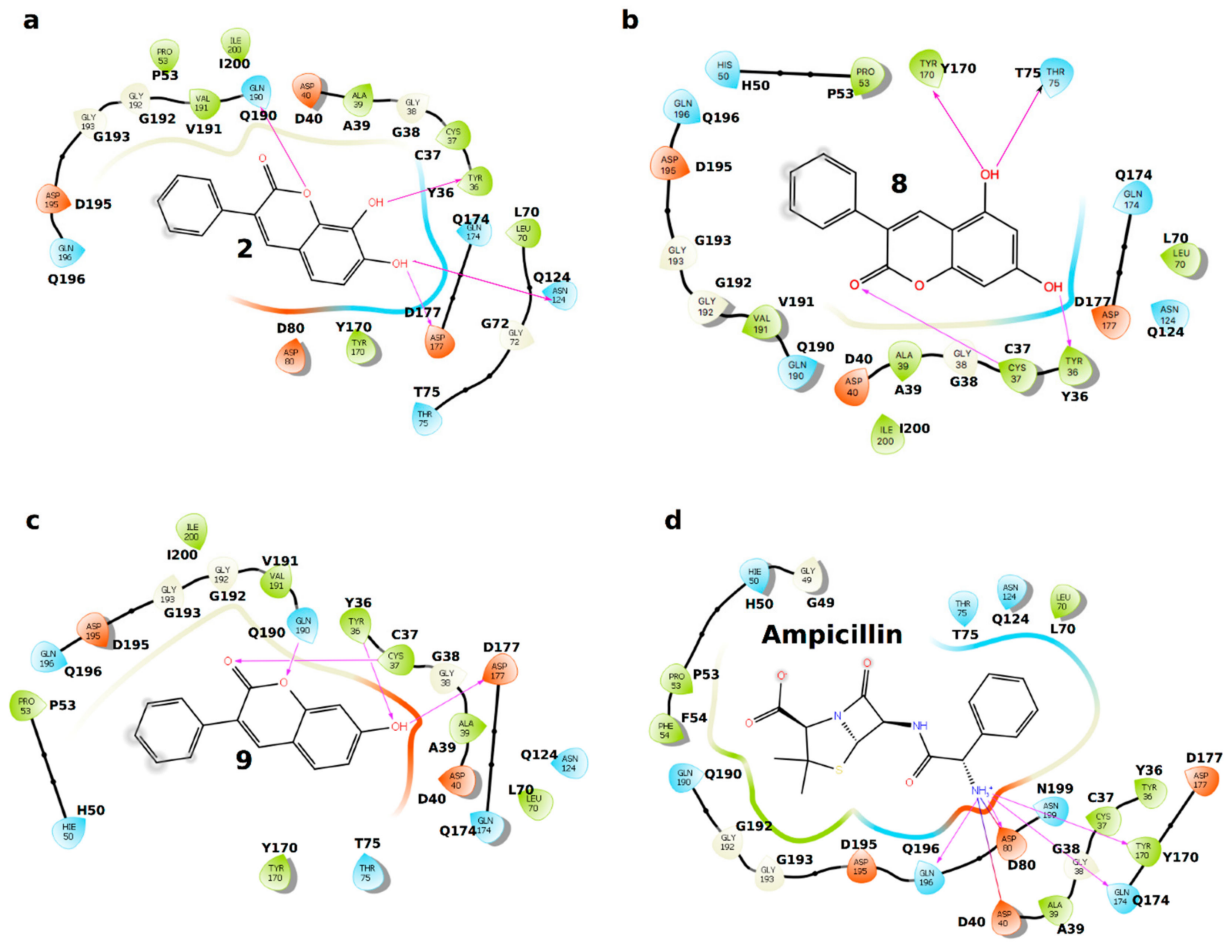
Interaction network between *S. aureus* tyrosyl-tRNA synthetase protein and the studied compounds. The negatively charged residues are shown in red, polar residues in cyan, and hydrophobic residues in parrot green. Hydrogen bond (H-bond) interactions are shown as pink arrow.

**Figure 3:**
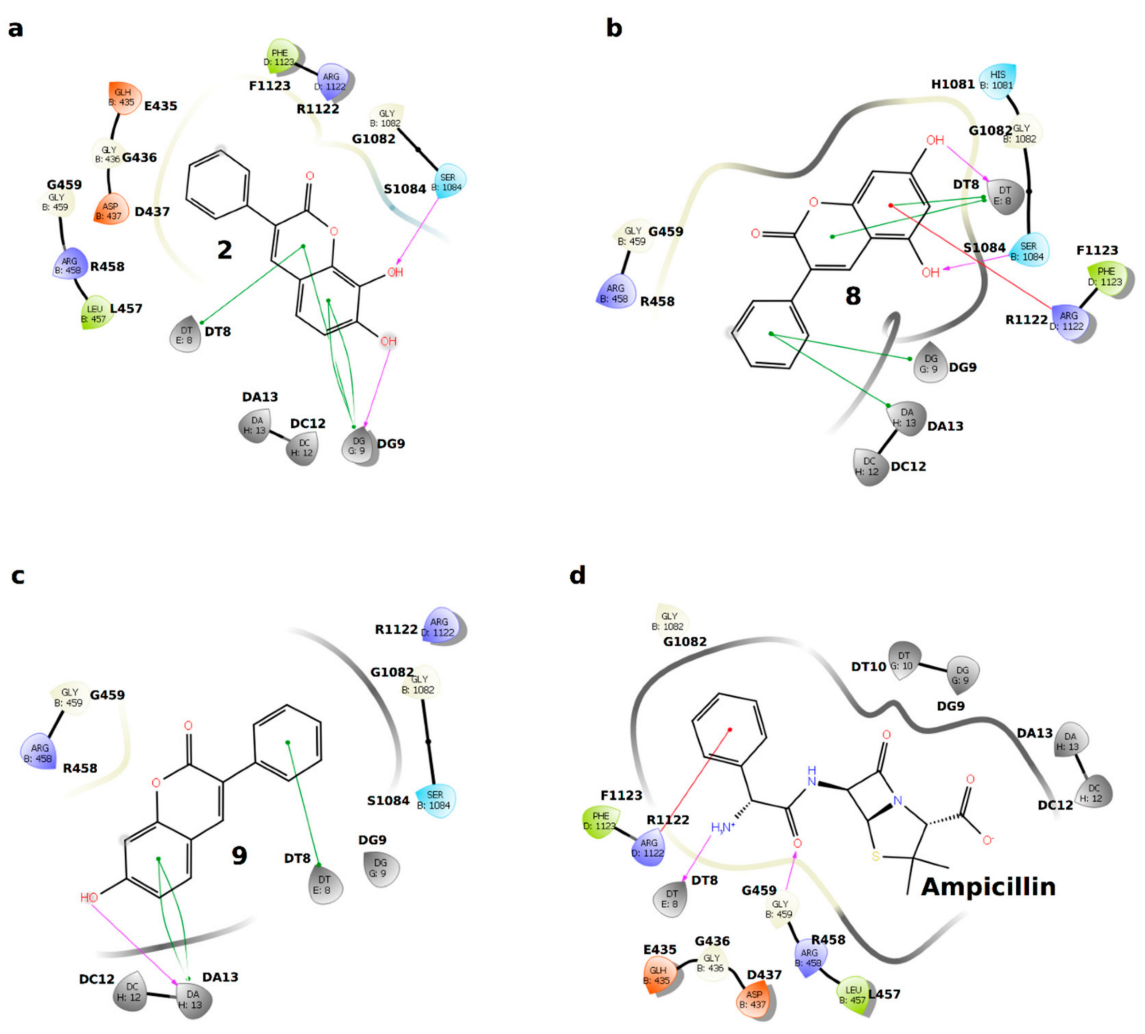
Interaction network between topoisomerase II DNA gyrase and the studied compounds. The protein residues with a negative charge are shown in red, positive charge in velvet, polar in cyan, and hydrophobic in parrot green. The DNA bases are shown in grey. The H-bond interactions are shown as pink arrow, pi–pi stacking as green line, and pi–cation as red line.

Molecular docking of compounds **2**, **8**, and **9** and ampicillin was performed to identify the binding sites on the structure of *S. aureus* tyrosyl-tRNA synthetase (Figure 2) and topoisomerase II DNA gyrase (Figure 3) proteins. Redocking of the cocrystal ligands was performed to validate the docking protocol. We found variation between 1 and 2 Å for the root mean square deviation (RMSD) values and a conserved binding pattern (Figure S1, Supplementary Materials).

Docking results against *S. aureus* tyrosyl-tRNA synthetase protein indicated a well-conserved binding region but with slightly different predicted best binding energy values. The best free binding energy was found for compound **8** (−9.2 kcal/mol), followed by compound **2** (−9.0 kcal/mol), and the lowest value was for compound **9** (−8.5 kcal/mol). In comparison to the other investigated compounds, ampicillin (reference molecule) displayed favorable binding energy value by ~2 kcal/mol. This aspect was consistent with a higher number of interactions noted for the ampicillin complex. The observed trend in the binding free energy was found to be consistent with the experimental inhibition trend for the molecules.

A very similar value of binding free energy (~7.0 kcal/mol) was found for compounds **2**, **8**, and **9** against topoisomerase II DNA gyrase. In all the investigated systems, we found aromatic–aromatic interactions involving the bases of DNA and the ligand. We noted significant changes in the ligand–receptor interaction network between the molecular systems. Compounds **2** and **8** displayed a higher number of hydrogen bond interactions and aromatic–aromatic interactions with respect to compound **9**. Furthermore, we found pi–cation interactions involving residue Arg-1122 only for compound **8** and ampicillin.

## 3. Materials and Methods

### 3.1. Chemistry

Compound **1** was obtained from Sigma-Aldrich (St Louis, Missouri, USA) and derivatives **2**–**10**, which are known, were prepared as described below and their purity determined by elemental (CHN) analysis. The NMR (^1^H and ^13^C) and mass spectral data as well as the melting point values of compounds **2**–**10** were found to be comparable to the literature data.

#### General Procedure for the Synthesis of Hydroxy-3-arylcoumarins (**2**–**10**)

1st step. Acetoxy intermediates were synthesized under anhydrous conditions using material previously dried at 60 °C for at least 12 h and at 300 °C for a few minutes immediately before use. A solution containing anhydrous CH_3_CO_2_K (2.94 mmol), arylacetic acid (1.67 mmol), and the corresponding hydroxybenzaldehyde (1.67 mmol) in Ac_2_O (1.2 mL) was refluxed for 16 h. The reaction mixture was cooled, neutralized with 10% aqueous NaHCO_3_, and extracted with EtOAc (3 × 30 mL). The organic layers were combined, washed with distilled water, dried (anhydrous Na_2_SO_4_), and evaporated under reduced pressure. The product was purified by recrystallization in EtOH and dried to afford the desired compound.

2nd step. Hydroxyl derivatives **2**–**10** were obtained by hydrolysis of their acetoxylated counterparts. The appropriate acetoxylated coumarin, mixed with 2N aqueous HCl and MeOH, was refluxed for 3 h. The resulting reaction mixture was cooled in an ice bath, and the reaction product, obtained as solid, was filtered, washed with cold distilled water, and dried under vacuum to afford the desired compound.

List of synthesized compounds: 7,8-dihydroxy-3-phenylcoumarin **2** [26], 7,8-dihydroxy-3-(3-hydroxyphenyl)coumarin **3** [28], 7,8-dihydroxy-3-(2-hydroxyphenyl)coumarin **4** [28], 3-(3,4-dihydroxyphenyl)-7,8-dihydroxycoumarin **5** [28], 7,8-dihydroxy-3-(thiophen-3-yl)coumarin **6** [26], 3-(4-bromothiophen-2-yl)-7,8-dihydroxycoumarin **7** [29], 5,7-dihydroxy-3-phenylcoumarin **8** [30], 7-hydroxy-3-phenylcoumarin **9** [31], 8-hydroxy-3-phenylcoumarin **10** [30].

### 3.2. Biological Studies

#### 3.2.1. Bacterial Cultures

The antibacterial activity of the tested compounds was evaluated using the following microbial strains: *Staphylococcus aureus* ATCC 25923, methicillin-resistant *S. aureus* TN2A (MRSA strain belonging to the collection of the Department of Medical Sciences and Public Health, University of Cagliari), *Bacillus cereus* (*B. cereus*) ATCC 11178, *Listeria monocytogenes* (*L. monocytogenes*) ATCC 19115, *Escherichia coli* (*E. coli*) ATCC 25922, *Salmonella enterica* serovar Enteritidis (*S.* Enteritidis) ATCC 13076, and *Pseudomonas aeruginosa* (*P. aeruginosa*) ATCC 27853. All bacterial strains were stored on nutrient broth (NB, Microbiol, Cagliari, Italy) plus 20% (*v/v*) glycerol at −20 °C. Before use, they were subcultured twice in the appropriate medium. MICs and minimum bactericidal concentrations (MBCs) of the compounds were determined by a broth microdilution method as previously described [32]. All tests were performed with NB, and the compounds were dissolved in DMSO (5% *v/v*). Serial doubling dilutions of each compound were performed in a 96-well microtiter plate in a final volume of 100 µL. The final concentrations of the compounds ranged from 7.8 to 500 µg/mL except for compound **8**, which was tested at concentrations ranging from 5.5 to 350 µg/mL. Overnight broth cultures were prepared in NB and adjusted so that the final concentration in each well following inoculation was approximately 5.0 × 10^5^ cfu/mL. The concentration of each inoculum was confirmed using viable counts on tryptic soy agar (TSA, Microbiol) plates. The controls included sterility of NB, sterility of the compounds, control culture (inoculum), and control DMSO to check the effect of solvent on the growth of microorganisms. Furthermore, ampicillin and gentamicin were used as positive control for Gram-positive and Gram-negative bacteria, respectively. The MICs and MBCs were determined after 24 h incubation of the plates at 37 °C. Microbial growth was indicated by the presence of turbidity and a “pellet” on the well bottom. MICs were determined as the first well, in ascending order, which did not produce a pellet. To confirm MICs and to establish MBCs, 10 µL of broth were removed from each well and inoculated on TSA plates. After incubation under the conditions described above, the number of surviving microorganisms was determined. The MIC was the lowest concentration, which resulted in a significant decrease in inoculum viability (> 90%), while the MBC was the concentration where 99.9% or more of the initial inoculum was killed. All tests were conducted in triplicate and with three replications, and the modal MIC and MBC values were selected.

#### 3.2.2. Antioxidant Activity

The total free radical-scavenging capacity of compounds was determined by ABTS [2,20-azinobis-(3-ethylbenzothiazoline-6-sulfonic acid)] method using 6-hydroxy-2,5,7,8-tetramethylchromane-2-carboxylic acid (Trolox) as standard, as previously described [33,34,35]. Briefly, the free radical ABTS was produced by reacting 7 mM ABTS with 2.45 mM potassium persulfate (final concentration) in aqueous solution and kept in the dark at room temperature for 24 h before use. Each compound (10 µL of an appropriate dilution) was added to 1 mL of ABTS, and the absorbance was recorded at 734 nm after 1 min incubation. Results are expressed as EC_50_ values (µM), calculated as concentration of compound that produces a 50% reduction in the original absorbance.

#### 3.2.3. Statistical Analyses

Statistical differences were evaluated using GraphPad Prism software version 8 (San Diego, CA, USA). Comparison between groups was assessed using one-way analysis of variance (one-way ANOVA) followed by Tukey’s multiple comparison test. The values with *p* < 0.001 were considered significant. Data are represented as mean ± SD of three independent experiments.

### 3.3. Computational Methodology

#### Docking Studies

The starting three-dimensional (3D) structure of *S. aureus* tyrosyl-tRNA synthetase (PDB id: 1JIJ) and topoisomerase II DNA gyrase (PDB id: 2XCT) were obtained from the Protein Data Bank (PDB) [36]. Ligand molecules were sketched using the Marvin JS tool of ChemAxon (http://chemaxon.com). The molecules were converted into 3D using open Babel software tool [37]. Before performing the docking protocol, chemically correct models of the ligands were generated using the ligprep module of Schrodinger, and the receptor structures were prepared using the Protein Preparation Wizard Module [38]. Molecular docking was carried out using the Glide ligand docking module (Schrödinger Release 2019-1: LigPrep, Schrödinger, LLC, New York, NY, 2019). Receptor grids were generated using the Glide receptor grid generation module. Grids were generated for the prepared proteins. For *S. aureus* gyrase complex, the grid was generated around ciprofloxacin, while for *S. aureus* TyrRS complex, the grid was generated around SB-239629 ligand. The boundary box was at default value, i.e., 14 Å × 14 Å × 14 Å, which was spacious enough to encompass the binding region. Further details of the protocol employed have been described previously [39]. Docking between the *S. aureus* tyrosyl-tRNA synthetase (PDB id: 1JIJ) protein structure and the ligands were also performed using COACH-D server [40], which employs the Autodock Vina algorithm [41], as described previously [42]. For comparison, docking of the ligands was also performed on higher-resolution structure of *S. aureus* tyrosyl-tRNA synthetase (PDB id: 1JIL) and topoisomerase II DNA gyrase (PDB id: 2XCS) (Figures S2 and S3, Supplementary Materials).

## 4. Conclusions

A family of 3-aryl/heteroarylcoumarins with structural features of flavonoids was synthesized and studied for their antibacterial and antioxidant profiles. 3-Phenylcoumarin derivative **8**, substituted with hydroxyl groups at the **5** and **7** positions, showed the highest antibacterial activity against a panel of Gram-positive pathogens, including an MRSA strain, as well as an interesting antioxidant profile. The presence of a phenyl ring at position 3 of the coumarin could be important as it is a structural feature of the most active compound within the series. This compound could be considered for its antibacterial potential and could be a valuable source for the design and development of new anti-infective compounds. As the most active compound for further studies, docking studies on two important targets were performed to elucidate a potential mechanism of action for this compound. Both tyrosyl-tRNA synthetase and topoisomerase II DNA gyrase from *S*. aureus were studied as potential targets, and a correlation between the observed inhibitory activity and the in silico molecular docking scores of the best compound **8** was obtained. The importance of the 3-phenyl ring of compound **8** was also corroborated by some of the aromatic–aromatic stacking interactions observed in the docking studies. Moreover, compound **8** also exhibited druglikeness properties, leading us to consider 3-phenyl hydroxycoumarins as a potential scaffold for improving antibacterial activity. Future studies will be carried out to evaluate the antibiofilm activity of this compound and to explore the biological activities of the metal complexes to find new compounds with increased antimicrobial potential and a broader spectrum activity.

## Figures and Tables

**Table:** (**a**)

Compounds	A. Gram-Positive Bacterial Strains							
	*S. aureus* ATCC 25923		*S. aureus* TN2A		*L. monocytogenes* ATCC 19115		*B. cereus* ATCC 11178	
	MIC	MBC	MIC	MBC	MIC	MBC	MIC	MBC
**1**	500	>500	500	>500	500	>500	500	>500
**2**	62.5	250	62.5	250	125	500	15.6	62.5
**3**	62.5	500	125	500	250	>500	62.5	250
**4**	125	500	250	>500	250	>500	62.5	250
**5**	125	500	125	500	250	>500	125	250
**6**	500	>500	500	>500	500	>500	250	500
**7**	>500	n.d	>500	n.d	>500	n.d	>500	n.d
**8**	11	87.5	22	87.5	44	350	11	44
**9**	>500	n.d	>500	n.d	>500	n.d	>500	n.d
**10**	>500	n.d	>500	n.d	>500	n.d	>500	n.d
Ampicillin ^a^	2.5	-	10	-	5	-	10	-

**Table:** (**b**)

Compounds	B. Gram-Negative Bacterial Strains					
	*E. coli* ATCC 25922		*S.* Enteritidis ATCC 13076		*P. aeruginosa* ATCC 27853	
	MIC	MBC	MIC	MBC	MIC	MBC
**1**	500	>500	500	>500	>500	n.d
**2**	500	>500	>500	n.d	>500	n.d
**3**	>500	n.d	>500	n.d	>500	n.d
**4**	>500	n.d	>500	n.d	>500	n.d
**5**	>500	>500	>500	>500	>500	n.d
**6**	500	>500	500	>500	>500	n.d
**7**	>500	n.d	>500	n.d	>500	n.d
**8**	>350	n.d	>350	n.d	>350	n.d
**9**	>500	n.d	>500	n.d	>500	n.d
**10**	>500	n.d	>500	n.d	>500	n.d
Gentamicin ^a^	5	-	5	-	5	-

^a^ Positive control. n.d: not determined.

**Table 2:** Antioxidant activity of the most active compounds evaluated by the ABTS scavenging method.

Compounds	EC_50_ (µM)
**2** ^^^	11.59 ± 0.39 ^a^
**3**	17.42 ± 0.42 ^b^
**4**	23.05 ± 0.43 ^c^
**5**	4.40 ± 0.07 ^d^
**8** ^^^	7.08 ± 0.04 ^e^
Trolox ^$^	13.0 ± 1.1 ^a^

The data are given as mean ± standard deviation (SD) of triplicate experiments. ^^^ Data previously described in reference [26]. ^$^ Positive control. Different letters denote statistically significant differences between compounds (*p* < 0.001).

**Table 3:** Molecular properties of the molecules under study. (**A**) Physicochemical properties and (**B)** pharmacokinetics.

**A. Physicochemical Properties**			
**Compounds**	**2 and 8**	**9**	**Ampicillin**
Molecular Formula	C_15_H_10_O_4_	C_15_H_10_O_3_	C_16_H_19_N_3_O_4_S
Molecular Weight (g/mol)	254.24	238.24	349.40
Rotatable bonds	1	1	5
H-bond acceptor atoms	4	3	5
H-bond donor atoms	2	1	3
Molar refractivity	71.97	69.94	92.56
Polar Surface area	70.67	50.44	138.03
Lipophilicity (consensus)	2.46	2.91	0.08
Water solubility	Soluble	Soluble	Very soluble
**B. Pharmacokinetics**			
Gastrointestinal absorption	High	High	Low
Blood–brain barrier permeation	Yes	Yes	No
P-glycoprotein substrate	No	No	No
Cytochrome P450 1A2 inhibitor	Yes	Yes	No
Cytochrome P450 2D6 inhibitor	No	No	No
Cytochrome P450 3A4 inhibitor	No	No	No
Druglikeness (Lipinski rule)	Yes	Yes	Yes

## Supplementary Materials

The following are available online at https://www.mdpi.com/1420-3049/24/15/2815/s1, Figure S1: Validation of docking protocol. Ligand superimposition: co-crystal and docked conformations. Figure S2: Docked conformation of the investigated ligands in (PDB id: 1JIL) Staphylococcus aureus tyrosyl-tRNA synthetase protein structure. Figure S3: Docked conformation of the investigated ligands in (PDB id: 2XCS) S. aureus Gyrase complex.

## Figures, Scheme and Tables

Table 1 Antibacterial activity of hydroxy-3-arylcoumarins against Gram-positive (**A**) and Gram-negative (**B**) bacterial strains. Minimum inhibitory concentration (MIC) and minimum bactericidal concentration (MBC) values are expressed in µg/mL.
