# Supplementary material for: Antibacterial Activity and Molecular Docking Studies of a Selected Series of Hydroxy-3-arylcoumarins
Source: Molecules. 2019 Aug 1;24(15):2815. doi: 10.3390/molecules24152815 (PMC6696357; doi:10.3390/molecules24152815)
Supplement: Supplementary file 1 [file molecules-24-02815-s001.pdf]

molecules

Supplementary Materials

Antibacterial Activity and Molecular Docking Studies of a Selected Series of Hydroxy-3-arylcoumarins

Maria Barbara Pisano [1,†], Amit Kumar [2,†], Rosaria Medda [3], Gianluca Gatto [2], Rajesh Pal [4], Antonella Fais [3,*], Benedetta Era [3,*], Sofia Cosentino [1], Eugenio Uriarte [5], Lourdes Santana [5], Francesca Pintus [3,#] and Maria João Matos [5,#]

[1] Department of Medical Sciences and Public Health, University of Cagliari, Cittadella Universitaria, 09042 Monserrato, Italy
[2] Department of Electrical and Electronic Engineering, University of Cagliari, Via Marengo 2, 09123 Cagliari, Italy
[3] Department of Sciences of Life and Environment, University of Cagliari, Cittadella Universitaria, 09042 Monserrato, Italy
[4] Department of Biomedical Sciences, University of Cagliari, Cittadella Universitaria, 09042 Monserrato, Italy
[5] Department of Organic Chemistry, University of Santiago de Compostela, 15782 Santiago de Compostela, Spain
* Correspondence: fais@unica.it (A.F.); era@unica.it (B.E.); Tel.: +390706754506 (A.F.); +390706753185 (B.E.)
† These authors contributed equally to this work
# These authors jointly supervised this work

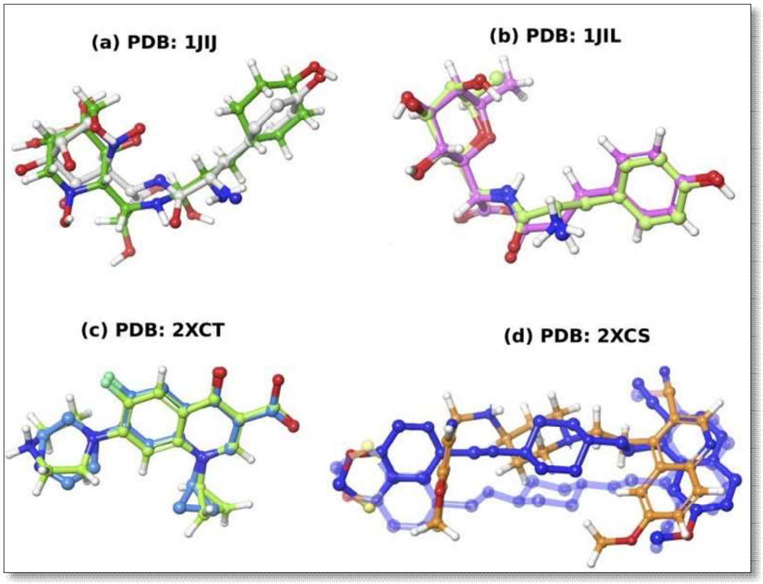

Figure S1. Validation of docking protocol. Ligand superimposition: co-crystal and docked conformations.

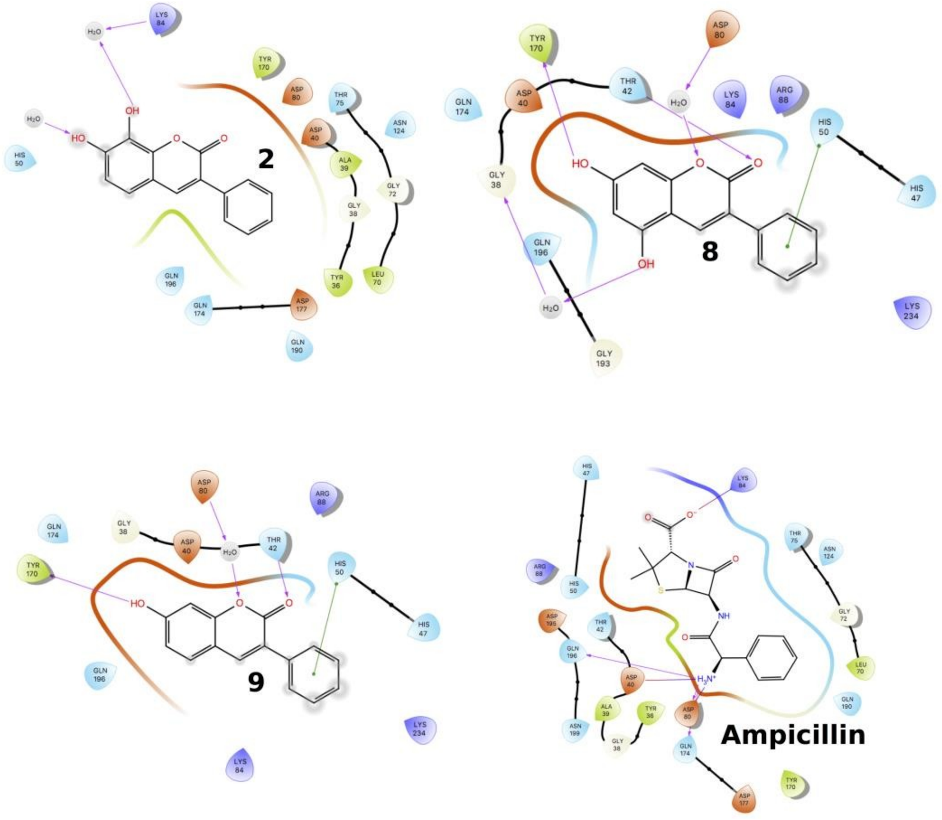

Figure S2. Docked conformation of the investigated ligands in (PDB id: 1JIL) Staphylococcus aureus tyrosyl-tRNA synthetase proteinstructure.

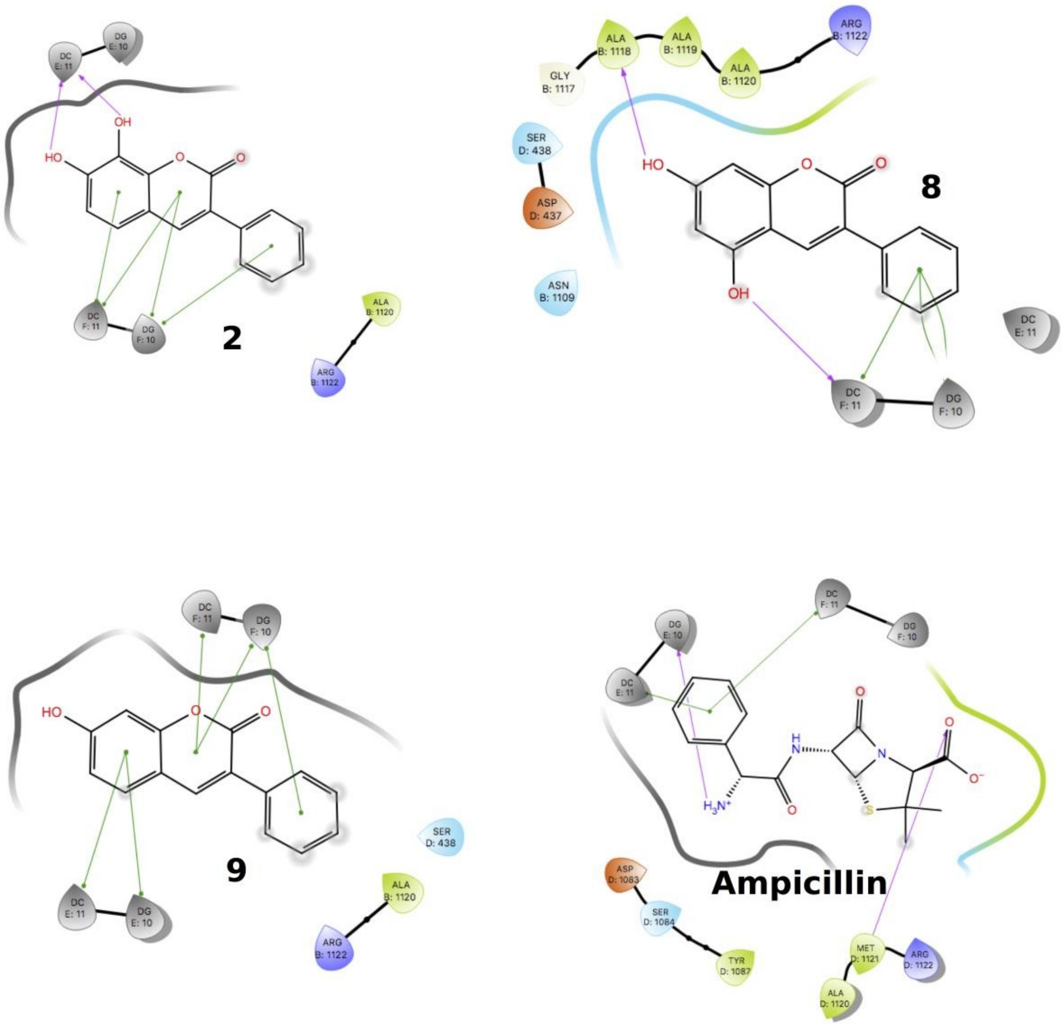

Figure S3. Docked conformation of the investigated ligands in (PDB id: 2XCS) S. aureus Gyrase complex.
